# Disease classification, diagnostic challenges, and evolving clinical trial design in MASLD

**DOI:** 10.1172/JCI189953

**Published:** 2025-05-15

**Authors:** Mette Munk Lauridsen, Kim Ravnskjaer, Lise Lotte Gluud, Arun J. Sanyal

**Affiliations:** 1 Stravitz-Sanyal Liver Institute, Department of Gastroenterology & Hepatology, Virginia Commonwealth University Medical Clinic, Richmond, Virginia, USA.; 2 University Hospital of Southern Denmark, Liver Research Group, Department of Gastroenterology and Hepatology, Esbjerg, Denmark.; 3 Department of Biochemistry and Molecular Biology, University of Southern Denmark, Odense, Denmark.; 4 Gastro Unit, Copenhagen University Hospital, Hvidovre, Denmark, and Department of Clinical Medicine, Faculty of Health and Medical Sciences, University of Copenhagen, Copenhagen, Denmark.

## Abstract

Metabolic dysfunction–associated steatotic liver disease (MASLD) diagnosis and management have evolved rapidly alongside the increasing prevalence of obesity and related complications. Hepatology has expanded its focus beyond late-stage cirrhosis and portal hypertension to earlier, complex MASLD cases in younger patients, necessitating closer collaboration with endocrinology. The renaming of nonalcoholic fatty liver disease (NAFLD) to MASLD reflects its pathophysiology, reduces stigma, and has prompted new research directions. Noninvasive tests such as liver stiffness measurement now play a crucial role in diagnosis, reducing reliance on invasive liver biopsies. However, advanced omics technologies, despite their potential to enhance diagnostic precision and patient stratification, remain underutilized in routine clinical practice. Behavioral factors, including posttraumatic stress disorder (PTSD) and lifestyle choices, influence disease outcomes and must be integrated into patient management strategies. Primary care settings are critical for early screening to prevent progression to advanced disease, yet sizable challenges remain in implementing effective screening protocols. This Review explores these evolving aspects of MASLD diagnosis and management, emphasizing the need for improved diagnostic tools, multidisciplinary collaboration, and holistic care approaches to address existing gaps and ensure comprehensive patient care across all healthcare levels.

## Introduction

The rise in obesity rates has caused the traditional hepatology outpatient clinic clientele of late-diagnosed, elderly patients with complications of cirrhosis and portal hypertension to be increasingly joined by early diagnosed, younger, obese, and multimorbid individuals with metabolic dysfunction–associated steatotic liver disease (MASLD). Patients with MASLD have the highest comorbidity burden, and the current nomenclature highlights the importance of these patients in the cirrhotic population, where they were not part of the development of management plans in a formal way. This shift not only bridges the gap between hepatology and endocrinology, but also underscores the need for refined disease classification and diagnostic strategies. The renaming of nonalcoholic fatty liver disease (NAFLD) to MASLD and the subsequent updates in diagnostic criteria have sparked considerable research and aim to align the disease nomenclature more closely with its pathophysiology and reduce stigma. For many clinicians, the shift from mostly managing complications to portal hypertension to managing the complex multidimensional metabolic and hepatic condition that make up MASLD poses a challenge and raises a multitude of questions. The clinical approach to MASLD is rapidly evolving due to a surge in research interest over the past decade, initially sparked by the simple recognition of obesity as a risk factor for liver disease and further fueled by massive interest from the pharmaceutical industry and new technological advances. The dynamic landscape of MASLD treatment underscores the need for continuous review of current and emerging trends. This Review does not merely summarize existing guidelines, but also promotes discussion on innovative patient-centered approaches in hepatology. It encompasses a forward-looking perspective that advocates for a comprehensive treatment paradigm, addressing both the physiological and mental health aspects of metabolic dysfunction. We aim to furnish a succinct synopsis of recent research and clinical advancements, underscoring progress in disease classification — including its notable shortcomings — as well as persistent diagnostic challenges and the structuring of clinical trials. The Review is designed to enlighten new hepatologists and all clinicians operating within the primary sector as well as those across various medical disciplines concerned with the health implications of metabolic dysfunction. Additionally, it seeks to provide translational and basic researchers with an updated perspective on the current state of clinical developments.

## MASLD disease classification

### New nomenclature and disease classification.

In late 2023, NAFLD was renamed MASLD, and the diagnostic criteria were updated. The nomenclature change was implemented following a modified Delphi process to minimize the stigma caused by the term “fatty” in NAFLD and address the irrationality of naming a disease after what doesn’t cause it (1). The term “fatty” in NAFLD was believed to stigmatize patients, especially in English-speaking nations, and its replacement with “steatotic” was meant to minimize stigma and create a better link to the pathophysiology. Further, the new nomenclature considers that liver disease can be multifactorial, e.g., caused by both alcohol and metabolic dysfunction, and as such, represents a more modern and holistic patient approach. Although slight, the change in diagnostic criteria spurred a massive research output trying to delineate how the new terminology affected the validity of prior findings made in the NAFLD era (2, 3). These reports found that NAFLD and MASLD patients are largely the same (4–6) and that noninvasive tests (NITs) such as liver stiffness measurement (LSM) keep their accuracy (7).

**Figure 1:**
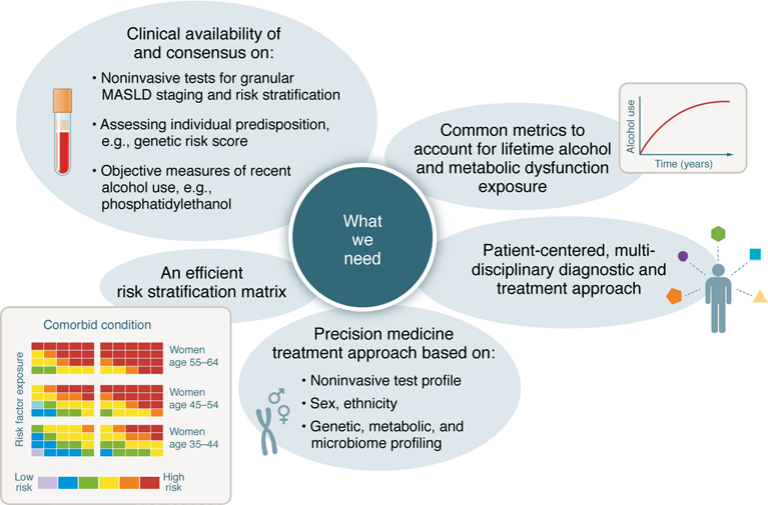
Outline of some of the desired components of future MASLD diagnosis, risk stratification, and management.

The recent initiative to refine disease classification through the introduction of a new nomenclature is an exemplary endeavor aimed at improving the precision of clinical diagnosis and enhancing the quality of research outcomes. Despite these advancements, it is imperative to continuously scrutinize and evolve the classification framework to better serve both clinical and research applications. The new (and the old) nomenclature is merely a crude classification, so aside from a focus on classification, clinicians should try to map the number of metabolic risk factors and duration of exposure and, similarly, lifetime total alcohol use, patterns of use, and types of alcohol consumed as these are dynamic modulators of liver disease progression and should be considered in this context. To this end, we still need a standard metric for measuring lifetime alcohol exposure (Figure 1) and perhaps a measure of metabolic dysfunction exposure.

Understanding the behavioral underpinnings of obesity and subsequently, MASLD and alcohol-related liver disease (ALD) is essential, as these diseases are profoundly shaped by behavior-regulated lifestyle choices. In this context, it is crucial to recognize that the politics of food availability and affordability across different nations plays a more pivotal role than individual lifestyle choices (8). Moreover, individual biological factors such as genetics and hormonal status influence one’s cardiometabolic risk in an obesogenic environment of low-quality foods and alcohol (9–11). Still, it is equally important to consider how individual life circumstances, mental habits, and competencies shape choices in environments dominated by disease-promoting foods and widely accessible alcohol (12). Conditions such as depression, anxiety, posttraumatic stress disorder (PTSD), eating disorders, impulsivity, and addictive behaviors are notably prevalent in obese patient groups and likely influence the onset and progression of liver diseases, as well as degrade quality of life (13–15). Therefore, these issues deserve greater focus from healthcare professionals and heightened scrutiny in scientific research, as their recognition and treatment could substantially enhance outcomes beyond the benefits of merely addressing obesity and its associated comorbidities (16, 17).

## Diagnostic challenges in primary care

About 30% of the global adult population is affected by MASLD (18, 19). The aim of screening at-risk individuals is the timely identification of patients with a high risk of adverse outcomes without overdiagnosis. Early diagnostics in MASLD is important as it allows for early implementation of interventions, including education and engagement of patients to prevent progression (20, 21). Lifestyle changes can revert early MASLD and prevent progression to clinically significant or advanced fibrosis. It also allows for monitoring and management of metabolic comorbidities, especially type 2 diabetes, and cardiovascular disease and risk factors, which can be treated effectively to improve long-term outcomes (22–24).

In the primary care setting, prioritizing sensitivity is recommended to ensure that patients with advanced fibrosis are not missed. This implies that ruling out advanced fibrosis is the task at hand. In that context, it is noteworthy that of the 30% of the population with MASLD, only a small fraction will have fibrosis, and an inherent issue with screening in a low-prevalence setting is that the positive predicted value will exceed 15%–17% even with sensitivity and specificity of 90%.

The initial recommended steps include calculating the fibrosis-4 index (FIB-4), which is based on age, aspartate aminotransferase (AST), alanine aminotransferase (ALT), and platelet counts, at risk populations. FIB-4 was designed to rule out advanced fibrosis in populations with a low prevalence of this phenotype. A FIB-4 value of less than 1.3 is recommended for this purpose. The FIB-4 value is impacted by age and for those above 65 years of age, a cutoff of 2 is recommended (25). FIB-4 is also robustly linked to the risk of clinical outcomes, which makes it even more useful as an initial screening test in primary care that should be used in those with metabolic risk factors (26). FIB-4 has a high sensitivity but modest specificity and in the presence of low pretest probability of advanced (F4) fibrosis has a negative predictive value over 90% to identify those with advanced fibrosis (19, 25, 27–30). It is, unfortunately, not designed to detect earlier stages of fibrosis, which is a concern, as medical treatment could eventually be considered in F2–F3 fibrosis (31). Nevertheless, implementation of FIB-4 in primary care could prove to be a turning point in hepatology, allowing us to move from a focus on symptom management in compensated or decompensated cirrhosis toward prevention of progression to cirrhosis and, with the advent of new treatments, also fibrosis reversal. Strengthening the collaboration and knowledge sharing between primary and secondary/tertiary care is crucial at this point if the aim is for primary care physicians to prioritize screening for MASLD as seriously as they do screening for other cardiometabolic risk factors.

### Enhanced risk stratification tools.

The FIB-4 has several limitations, including a poor positive predictive utility in populations with a low prevalence of advanced fibrosis (32). Further, the acceptance of steatotic liver disease across a spectrum from a purely metabolic to alcohol-driven process will require tools for risk assessment across the full expanded etiological spectrum of disease. Alcohol particularly increases AST and may lead to erroneous assessment of advanced fibrosis. Accurate assessment and anticipation of the progression from MASLD or metabolic dysfunction and alcohol-related liver disease (MetALD) to hepatic decompensation are critical for prioritizing patient care across all levels (33). Development of such tools in primary care is now a major public health need. Drawing on successful models from cardiology, the development of a similar risk stratification framework for MASLD could enhance referral precision in primary care. This framework would integrate reliable NITs like FIB-4 with factors that exacerbate MASLD risk — such as type 2 diabetes, male sex, age over 50, postmenopausal status in females, hypertension, dyslipidemia, and abdominal obesity. Additionally, emerging plasma and composite NITs, which are closely aligned with liver pathophysiology and specific genetic markers such as *PNPLA3*, promise to further refine this approach by providing personalized risk assessments grounded in pathophysiological insights (34, 35). This precision is crucial given the vast number of individuals at risk and the evolving array of targeted treatment options.

## Diagnostic challenges in secondary and tertiary care

Whereas the main objective in primary care is to rule out clinically significant fibrosis and focus on the management of the metabolic root causes of MASLD, the main objectives in secondary care are to confirm the risk strata for the patient and implement risk-based management strategies for more advanced disease. Advanced fibrosis is the main prognostic characteristic of MASLD and requires a treatment and monitoring plan (19, 25, 27–30), and fibrotic metabolic dysfunction-associated steatohepatitis (MASH) should give rise to considerations of resmetirome treatment (only in the US).

As such, the initial workup after a referral from primary care entails second-line NITs serving to identify false positives from the cohort of patients with FIB-4 above the threshold. Depending on availability, the second-line NITs are blood tests such as the enhanced liver fibrosis (ELF) test (hyaluronic acid, TIMP-1, PIIINP) followed by a confirmatory LSM or LSM alone. In a mixed population of ALD, MetALD, and MASLD patients, an ELF test in cases with indeterminate FIB-4 reduced false positives to 8% and resulted in the correct classification in 88% of cases (32). LSM has, in several studies, proved able to identify patients with 2 or more fibrosis as a stand-alone test and as a second-line NIT following FIB-4, making it the noninvasive gold standard (36–39). LSM is also a prognostic in MASLD: in patients with FIB-4 greater than 1.30, LSM 8.0–12.0 kPa and greater than 12.0 kPa, this is associated with an adjusted hazard ratio for a liver-related event of 3.8 and 12.4, respectively (40). Likewise, a change in LSM at retesting is associated with the risk of liver-related events (LRE) and is a noninvasive surrogate for clinical outcomes in patients with MASLD (36).

The drawbacks of LSM using e.g., FibroScan, are that obesity and increased waist circumference can affect measurements (41). Further, FibroScan (EchoSense), the first LSM device available, has largely monopolized the market with prices up to $170,000, making it unfeasible for point-of-care population-based screening. For both LSM and the ELF test, inflammation and comorbidities such as kidney disease can lead to misclassification of disease severity (42, 43). These tests also have lower accuracy for diagnosing the intermediate levels of fibrosis (F2–F3), which are potentially eligible for treatment (44, 45).

The simultaneous use of LSM with other NITs has also been examined and led to the Agile 3+, Agile 4 (LSM, AST, ALT, platelets, sex, diabetes, age), and FAST scores (LSM, CAP, AST) (46, 47). A recent multicenter validation study published in JAMA, including data from more than 16,000 at-risk individuals with vibration controlled transient elastography (VCTE) and blood sampling and an approximately 4.5-year follow-up, found that 1.9% developed LREs. The Agile scores were excellent at predicting these events, with an area under the receiver operating characteristic (AUROC) of 0.90 (36). The scores outperformed VCTE alone and even histological evaluations. This simultaneous test strategy is not suitable for use in primary care due to the limited availability of LSM. Therefore, the two- or three-tier sequential testing strategy currently dominates.

Another crucial diagnostic challenge that takes place in secondary care centers is the exclusion of dual pathology, especially for children and young adults (25).

## Improving diagnostics in secondary and tertiary care

### NITs for diagnosing MASH.

While liver fibrosis can be assessed noninvasively with good accuracy, liver biopsies remain the only diagnostic modality for MASH and, though effective, highlight a gap in MASH management due to their invasiveness. The discovery of MASH-specific NITs such as NIS2+ and Trem2 is promising (35, 48–51). These NITs are pivotal for detecting at-risk MASH, as defined by a NAFLD activity score (NAS) of 4 or more and fibrosis stage of 2 or greater. Their integration into clinical practice could allow for earlier and more precise interventions and ultimately improve patient outcomes.

### Comprehensive care approach.

**Figure 2:**
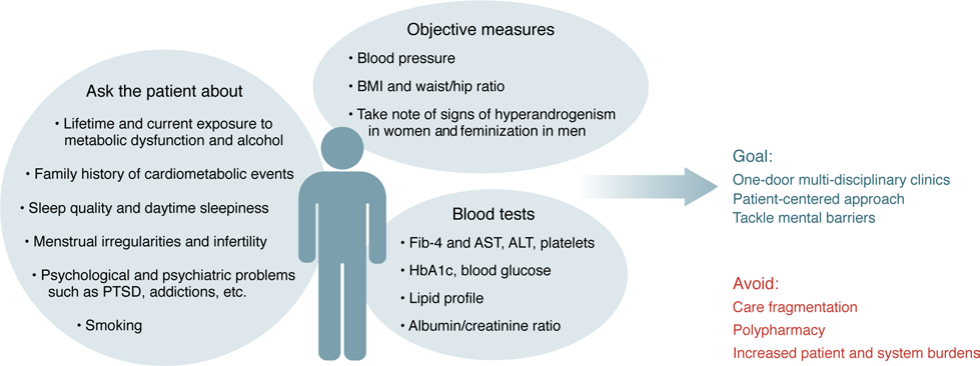
Basic assessment of patients at risk of metabolic dysfunction in primary and secondary care beyond the liver. The suggested holistic evaluation includes the cardiometabolic risk factors in the Framingham Risk Score and ASCVD. Some goals for patient care and suggestions on what to avoid are provided.

Adopting a holistic approach to diagnostics in MASLD is crucial due to the interconnected nature with metabolic syndrome, obesity, hypertension, dyslipidemia, and insulin resistance (25). Prioritizing screenings for type 2 diabetes and cardiovascular risk is crucial. Additionally, it is important to assess for conditions like sleep apnea, polycystic ovarian syndrome (PCOS), chronic kidney disease (CKD), and mental health issues, which are prevalent among patients with metabolic dysfunction. Recent advancements in the prevention and treatment of these conditions underscore the importance of their early detection to prevent detrimental outcomes (52–55). A comprehensive diagnostic approach ensures screening for diabetes with HbA1c, assessment of blood pressure, and lipid profile (cholesterol levels, including LDL, HDL, and triglycerides) in addition to risk scores like the atherosclerotic cardiovascular disease risk score (ASCVD) and the AHA PREVENT risk score (56–58). A rough screening for sleep apnea and PCOS could be done by simply inquiring about daytime sleepiness, menstrual irregularities, and infertility and looking for signs of hyperandrogenism, e.g., hirsutism or acne (59, 60). Screening for CKD with serum creatinine and urine albumin-to-creatinine ratio (ACR) to detect albuminuria may also be considered (61). To implement screening for additional metabolic complications in patients with MASLD, coordinated management across specialties are needed. Multidisciplinary teams, including primary care physicians, endocrinologists, cardiologists, and nephrologists, are important to ensure comprehensive care to reduce the risk of complications and improve overall health outcomes (28, 62–64). This is only feasible in resource-rich environments. In other areas, this will require a retraining of the work force to enable and empower them to engage in such holistic assessment and care delivery. Figure 2 illustrates components of a basic assessment of patients at risk of metabolic dysfunction beyond the liver.

## The future of MASLD diagnostics

Despite the proliferation of diagnostic modalities, the accessibility of these technologies remains limited, especially in resource-constrained environments. There is a critical need for point-of-care diagnostics that can be broadly distributed, ensuring that MASLD diagnosis is not confined to regions with access to advanced medical care. Looking forward, it is crucial to close this disparity, ensuring that all patients and healthcare systems, regardless of their economic status, have access to accurate and timely diagnosis. This challenge highlights the necessity of developing accessible, cost-effective diagnostic tools that can deliver advanced healthcare globally. With the awareness that advanced omics technologies will not be clinically available in most regions any time soon, we here discuss the potential and limitations of omics strategies in MASLD as diagnostic tools and as tools for innovation that can aid the development of accurate point-of-care diagnostics.

Rapidly evolving technological and computational capabilities have spurred the use of omics strategies for the discovery of liver disease biomarkers for patient risk stratification reviewed previously (65). The sensitivity and throughput of genomics, epigenomics, transcriptomics, proteomics, and metabolomics analyses have increased immensely, and novel single-cell and spatially resolved assays promise entirely new levels of insight (66–72). Despite the indisputable value of these omics technologies in biomarker discovery, their application in the clinic remains limited.

Recent progress in omics-guided biomarker panel development highlights that integration of complementary modalities may enhance diagnostic precision and risk stratification. The application of multi-omics in MASLD diagnostics and in the development of composite biomarker panels hence holds promise for advancing both diagnostics and personalized treatment.

### Multi-omics in noninvasive diagnostics.

Individual omics technologies such as transcriptomics (73–79), proteomics (80–85), or metabolomics (86–89) have been widely used to identify molecular signatures in peripheral blood for noninvasive biomarker discovery. These include circulating protein markers of fibrosis stage (AKR1B10) (73, 82), GDF15 (73), IGFBP7 (77), SEMA4D (77), SSC5D (77), SMOC2 (76), ADAMTSL2 (50), C7 (83), ICAM1 (83), ALDOB (83, 84), LGALS3BP (84)) or lobular inflammation (TREM2) (50, 78, 79), which may eventually serve in lower-plex biomarker panels more widely applicable in the clinic. In a display of its strengths, integration of different omics modalities was used for noninvasive detection of hepatocellular carcinoma (89). Composite signatures reflect cellular and extracellular processes typical of different disease aspects. Use of multi-omics also in MASLD diagnostics could hence ease interpretation, enhance accuracy, and provide dynamic insights, helping to stratify patients by disease risk (90–94). Integration with genetic information would further strengthen these aspects by exposing interactions between genetic traits and metabolic phenotypes and mapping regulatory nodes of molecular networks that shape disease trajectories. For a general discussion of multiomics approaches in noninvasive disease diagnostics, please refer to a previous publication (95).

### Liver biopsy–based multi-omics approaches.

Histopathological scoring of liver biopsies is still the gold standard for diagnosis of liver disease despite recognized shortcomings such as sampling bias and interassessor variability (96, 97). Multiplexed spatial profiling of transcripts, proteins, or metabolites in the biopsies would offer a less biased and more complete view of tissue processes predictive of disease risk and progression. Multi-omics has been applied experimentally to patient liver biopsies and delivered diagnostic and prognostic insights into various pathologies (98–101). Polymorphisms in liver disease–risk genes such as *PNPLA3* and *TM6SF2* have been linked directly to liver metabolism (102, 103). Genome-wide variant calling combined with spatial omics would better capture disease endotype, stage, and further trajectory. Deep learning–assisted pattern recognition and dimensional reduction of the rich data could further provide clinically useful scoring systems to complement current semiquantitative, histological assessments. While spatial omics technologies per se are still exploratory and unfeasible in most clinical settings, they too become cheaper and relevant to tertiary care centers. In parallel, artificial intelligence–based models are being developed to improve diagnostic accuracy of current and new staining methods agnostic to individual molecular species (97, 104).

### Challenges to multi-omics strategies.

(a) A first limitation in translating molecular profiles to disease risk is the availability of well-characterized patients in prospective studies for training and validation. These patients should represent the wider global population in terms of ethnicities, ages, sexes, medications, and cultural practices. Further, standardized study designs with representative (105) patient cohorts should ensure better reproducibility across laboratories. (b) Standardization of sample collection and analyses on well-preserved biopsies and plasma samples is critical for all aspects, from the generation of training data from multicenter cohorts to the practical implementation in the clinic. Subtle variation in sample collection, handling, and storage, not to mention diurnal variances, can reduce the repeatability and reproducibility of findings. (c) Costs and technical demands of multi-omics platforms limit their adoption in clinical settings. Future efforts should focus on developing cost-effective, robust methodologies and computational models to streamline data integration (106) and ensure clinical applicability. With robust feature selection algorithms, focused biomarker panels will serve as good proxies in healthcare settings where multi-omics analysis is not feasible. (d) Widespread application of deep learning–based approaches to multi-omics data may uncouple identification of molecular biomarkers from biological understanding. Interpretability in the biomarker selection process will facilitate efforts to relate molecular signatures to liver biology and help elucidate new avenues for disease intervention. (e) Regulatory approval is a bottleneck for (multi)omics-based biomarker implementation in the clinic. The path to approval requires attention to clinical needs and cohort distribution already in the discovery study design and demands careful adherence to regulatory requirements in subsequent validation studies (65, 107).

As challenges are overcome, multi-omics strategies in liver disease diagnostics hold tremendous promise for improving early detection, patient stratification, and effective personalized care.

## Evolving clinical trial design

### Challenges in trial design.

Developing clinical trials for MASLD involves navigating a series of intricate challenges. These challenges are primarily rooted in the complex pathophysiology of MASLD and the extensive variability in disease progression influenced by cohort-specific and individual-specific factors. One of the most critical obstacles in these trial designs is the lack of universally accepted, robust, noninvasive biomarkers of the studied outcome that can precisely monitor disease progression and effectively measure responses to therapeutic interventions (108). As discussed above, LSM offers the best proxy for noninvasively assessing fibrosis progression and regression; however, its dependence on highly trained personnel and costly equipment renders it less feasible for large-scale population-based trials (28, 109). Furthermore, a reliable biomarker for monitoring MASH specifically is still notably absent. This gap necessitates continued reliance on histological evaluations and lengthy observational periods to detect the onset of liver-related events, considered reliable endpoints to demonstrate clinical benefits. Such reliance profoundly complicates trial logistics, extending the duration and escalating costs, thereby placing additional burdens on study participants. Additionally, the complexity of MASLD’s pathophysiology, characterized by dynamic interactions among metabolic dysfunctions, genetic predispositions, and lifestyle factors, poses considerable challenges in stratifying participants and interpreting trial outcomes. These complexities highlight the urgent need for innovative approaches in clinical trial design and therapeutic strategies to enhance patient outcomes in MASLD. Ongoing advancements in this field are crucial. They will enable clinical scientists and the pharmaceutical industry to develop faster and more efficient clinical trials with more precise endpoints than those provided by traditional liver biopsy. Aided by the constant discovery of novel biomarkers and new technologies, the field is moving toward developing a circulating “liquid biopsy” strategy (cf. below) (110, 111). Also, new ultrasound- and MRI-based imaging technologies are emerging and could serve as noninvasive endpoints in clinical trials, although these are still limited by issues on availability (109).

## Innovations in tools and clinical trial design

### Precision medicine approaches.

Adoption of precision medicine strategies utilizing genetic, metabolic, and microbiome profiling aim to stratify patients based on their risk of progression and response to specific therapies (Figure 1) (112, 113). This stratification could enhance the efficacy of trials by targeting subgroups most likely to benefit from a given intervention. For example, *PNPLA3* polymorphisms, associated with increased fat accumulation and fibrosis risk, could be considered a personalized stratification tool in MASLD trials (114).

### Liquid biopsy — the surrogate endpoint of the future.

A “liquid liver biopsy” is an emerging concept in the field that refers to using blood tests to analyze biomarker panels that can provide information about liver disease features and their regression or progression in even greater detail than traditional tissue biopsies would (115, 116). This approach utilizes circulating biomarkers such as proteins, DNA methylation profiles, microRNAs, and extracellular vesicles. The advantages of a liquid liver biopsy, apart from its noninvasive nature, include the capability for real-time disease monitoring and broader accessibility compared with surgical biopsies.

### Adaptive trial designs.

Adaptive trial designs, which allow for modifications based on interim results, are gaining traction in MASLD research (117). These designs can include: (a) adaptive randomization, which implies adjusting the allocation ratio between experimental and control arms as the trial progresses, increasingly assigning more patients to the arm showing better outcomes. This approach dynamically refines patient distribution based on interim results to enhance the study’s overall efficacy. (b) Adaptive dose adjustments involve modifying the dosage of a drug within the trial based on interim data regarding its efficacy and safety. This strategy allows researchers to optimize the therapeutic effect of the drug while minimizing adverse effects, thereby tailoring treatment to achieve the best possible outcomes for participants. This method ensures that the trial can respond to real-time data and adjust the dosing regimen accordingly to meet the specific needs of the study. (c) Early termination for futility or efficacy, which refers to the ability to stop a clinical trial prematurely based on interim data analyses. This approach helps conserve resources and reduces patient exposure to treatments that may be ineffective or harmful. If the interim results show that the experimental treatment is unlikely to achieve the desired efficacy, the trial can be stopped for futility. On the other hand, if the data demonstrate significant benefits that exceed predefined thresholds, the trial may be halted early for efficacy, enabling quicker access to the treatment for a wider patient population.

The flexibility of adaptive trials can accelerate the development of effective therapies while conserving resources by discontinuing ineffective ones earlier.

### Integrated development programs for multiorgan benefits.

As our understanding of pathogenic mechanisms deepens, it is imperative that clinical trial designs evolve alongside. This involves integrating trials that test therapies across multiple chronic diseases with shared pathophysiology simultaneously within unique development programs. Initially, SGLT-2 inhibitors and GLP-1 receptor agonists were approved solely for glucose reduction, yet subsequent approvals were granted for cardiac, renal, and weight loss benefits (115). This paradigm shift suggests that future trials should assess multiorgan benefits using tailored endpoints for comprehensive regulatory approval, reflecting a more holistic approach to disease management.

Adaptive trial design and integrated development programs will likely speed up trials and the subsequent approval process, which is mainly positive. Conditional drug approvals are designed to provide early access to promising new therapies based on preliminary evidence, usually from phase I or phase II trials. An example in MASLD drug research is the phase 3 placebo-controlled MAESTRO trials, which resulted in conditional approval of resmetirome by the US FDA for treating adults with MASH and moderate-to-advanced fibrosis (116). However, there is an ongoing debate about whether conditional approvals might compromise the completion of full-scale outcome studies (phase III trials), as drugs are already on the market and generating revenue. Additionally, recruiting participants for these trials becomes more challenging when a therapy is available outside of the study setting. To support the full approval process, regulators can enforce strict timelines for confirmatory trials and require robust postmarketing surveillance to monitor the drug’s effectiveness and safety (116). Adaptive trial designs also play a role, allowing modifications based on interim data to maintain trial integrity.

## Collaborative approaches

The future of MASLD care involves multidisciplinary teams and collaborations among academia, industry, and regulatory bodies to standardize protocols, improve outcomes, and address unmet clinical needs.

## Conclusion

In conclusion, the evolving understanding and management of MASLD mark a critical turning point in hepatology. The renaming from NAFLD to MASLD reflects a broader, more holistic approach to liver disease, emphasizing the complex interplay of metabolic factors. While vital advancements have been made in disease classification and noninvasive diagnostic methods, critical challenges remain, particularly in accurate risk stratification and the detection of intermediate fibrosis stages. Looking forward, innovations in clinical trial design — especially adaptive trials and liquid biopsy techniques — offer the potential to streamline therapeutic development. Continued collaboration among clinicians, researchers, and the pharmaceutical industry will be essential to realize these advancements, ultimately improving outcomes for patients with MASLD.

## Author contributions

MML wrote the manuscript outline, introduction, disease classification, evolving clinical trial design, and figures. LLG wrote the diagnostic challenges. KR wrote the omics approaches, AJS supervised the project, made manuscript adjustments, and wrote the summary and concluding remarks.

## Version 1 05/15/2025

Electronic publication
